# CT imaging of myocardial viability: experimental and clinical evidence

**Published:** 2007-07

**Authors:** Andreas H Mahnken, Georg Mühlenbruch, Rolf W. Günther, Joachim E. Wildberger

**Affiliations:** Department of Diagnostic Radiology University Hospital, RWTH Aachen University, Germany; Applied Medical Engineering, Helmholtz Institute, RWTH Aachen University, Germany; Department of Diagnostic Radiology University Hospital, RWTH Aachen University, Germany; Department of Diagnostic Radiology University Hospital, RWTH Aachen University, Germany; Department of Diagnostic Radiology University Hospital, RWTH Aachen University, Germany

## Abstract

**Summary:**

Over the last decade, imaging of myocardial viability has become a well-established indication in patients suffering from myocardial infarction. Myocardial viability imaging is routinely performed using ^18^F-fluorodeoxyglucose positron emission tomography, single-photon emission computed tomography or magnetic resonance imaging. Only recently have several multi-slice spiral computed tomography (MSCT) techniques been evaluated for visualisation of myocardial infarction. This review describes the different concepts of MSCT viability imaging. MSCT assessment of myocardial morphology, myocardial perfusion imaging and delayed myocardial contrast enhancement are introduced, with the latter evolving as the key concept of MSCT viability imaging. Clinical relevance of the different MSCT techniques is described.

## Summary

During the last decade, great advances in cardiac imaging have been achieved. Magnetic resonance (MR) imaging as well as multi-slice spiral computed tomography (MSCT) have become routine tools for cardiac imaging. One of the most relevant clinical applications is visualisation of myocardial viability. As dysfunctional but viable myocardium has the potential for functional recovery after reperfusion,^1^ non-invasive assessment of myocardial viability allows for selecting patients who will benefit from a revascularisation therapy. This is an appealing idea, as revascularisation by percutaneous coronary angioplasty or coronary artery bypass grafting is cost intensive and still associated with relevant morbidity and mortality.

Moreover, the extent and degree of myocardial injury determines patient outcome and survival.^2^ After revascularisation, the annual mortality rate in patients with viable myocardium is half as high as in patients without viable myocardium. In patients with viable myocardium, medical treatment alone results in a five-fold higher annual mortality rate when compared with revascularisation.^3^

The transmural extent of infarction is also of crucial importance, as this determines outcome. The global left ventricular systolic function of patients who had successful reperfusion of their first acute myocardial infarction, resulting in necrosis of less then 25% of wall thickness, improved in 67% of patients. If the extent of irreversible myocardial damage was limited to less than 50% of myocardial thickness, regional left ventricular function improved in 56% of patients.^4^ With a transmural infarct size of more than 75%, functional recovery is extremely rare. Correspondingly, in patients with chronic, stable coronary artery disease and impaired left ventricular function, recovery of function after bypass grafting is rare when the transmural extent of scar tissue exceeds 75%.^5^

Therefore, assessment of myocardial viability with delayed contrast-enhanced MR imaging proved essential for risk stratification and treatment planning. In addition, there is only limited time before chronically hypoperfused myocardium becomes irreversibly damaged.^6^

In clinical routine, several techniques, including low-dose dobutamine stress echocardiography, single-photon emission computed tomography (SPECT), ^18^F-fluorodeoxyglucose positron emission tomography (PET) and MR imaging have been established for the assessment of myocardial viability. Except for MR imaging, these techniques do not allow differentiation between transmural and non-transmural infarction. MR imaging not only allows for detailed visualisation of viable and nonviable myocardium but also assessment of contractile function. Therefore, MR imaging has evolved as the gold standard for the assessment of myocardial viability.^7^

With the introduction of MSCT in cardiac imaging, a new modality entered the ring of myocardial viability imaging. Only recently have promising results been published, proving MSCT capable of differentiating viable from non-viable myocardium and having the ability to assess the transmural extent of myocardial infarction.^8^ This article introduces the pathophysiological basics and reviews the current state of CT imaging of the ischaemic myocardium.

## Ischaemic injury

Ischaemic injury of the myocardium can be differentiated as reversible and irreversible, as well as in acute and chronic conditions (Table 1).

**Table 1:** Classification Of Ischaemic Injury

	*Acute*	*Chronic*
Reversible	Stunning	Hibernation
Irreversible	Acute myocardial infarction	Chronic myocardial infarction

## Reversible – acute

Single or repeated short periods of myocardial ischaemia may result in myocardial stunning,^9^ a post-ischaemic dysfunctional state of the myocardium that persists even if the coronary flow is restored.^10^ Typical causes for stunning are exercise-induced ischaemia, heart transplantation, acute myocardial ischaemia and infarction dysfunction.^6^ Recovery from stunning seems to be related to repair by *de-novo* synthesis of proteins.^11^

## Reversible – chronic

Hibernating myocardium is characterised as viable but nonfunctional myocardium with chronically impaired regional blood flow.^12^ Hibernating myocardium is typically found in patients with haemodynamically relevant multi-vessel disease. Contractile function is diminished as a result of reduced myocyte metabolism in reaction to prolonged perfusion impairment. To some degree, function can be recovered with revascularisation.^13^

## Irreversible – acute

The loss of the cell membrane integrity marks the point of cell necrosis and irreversible myocardial infarction. In necrotic myocytes, the intracellular space is accessible to extracellular contrast media. In addition, cytokine-mediated interstitial oedema increases the distribution volume for contrast material.

In acute myocardial infarction, not all cells die simultaneously. 14 Instead, myocyte necrosis starts in the subendocardial layer of the myocardium and with prolonged ischaemia, myocyte death spreads like a wave front across the myocardium. This is due to the fact that at rest, most of the myocardial thickening occurs within the endocardial half of the myocardium.^15^ Consequently, the subendocardial myocardium has the highest energy demand. The extent of necrosis is a function of the occluded vessel territory, which determines the lateral boundaries and the duration of ischaemia, which, in turn determines the transmural extent of infarction. If the coronary arteries are reperfused before transmural infarction has developed, myocardium at risk in the mid-myocardial and subepicardial layer can be salvaged.^5^

Restoration of blood flow in the coronary arteries does not necessarily result in microvascular flow restoration;^16^ a so-called microvascular obstruction or no-reflow. Microvascular obstruction is correlated with an increased cardiac mortality in acute myocardial infarction and a reduced long-term prognosis.^17^

## Irreversible – chronic

Within 72 hours of acute myocardial infarction, thinning of the necrotic myocardium can be observed. Simultaneously, its endocardial surface area increases due to the reduced tensile strength of the infarcted tissue. Within six weeks, the necrotic myocardium is replaced by scar tissue that is markedly thinner than healthy myocardium. The time course of this remodelling process is influenced by several factors such as the severity of the underlying disease or secondary events.^18^

## MSCT assessment of myocardial viability

Up to now, MR imaging has been considered as the gold standard for viability imaging. There are some limitations, however, to cardiac MR imaging. For patients with implantable devices such as pacemakers and defibrillators, alternative techniques are needed to evaluate myocardial viability.

While coronary calcium scoring and coronary angiography are currently the key applications of cardiac MSCT, it also permits evaluation of the cardiac veins,^19^ myocardial perfusion, heart valves, ventricular volumes including ejection fraction, wall thickness, thickening and motion, and last but not least, myocardial viability.^20^ However, cardiac MSCT is coupled with a relevant radiation exposure. For 64-slice CT coronary angiography, dose values of up to 15.2 mSv for males and 21.4 mSv for females have been reported.^21^,^22^ Applying optimised examination protocols, the radiation exposure in late-phase MSCT is significantly lower, with approximately 2.8 mSv in male and 3.8 mSv in female patients.^8^ To reduce radiation exposure, several techniques including body weight-adapted tube current time settings and ECG-dependent tube current modulation may be used. The latter allows a dose reduction of up to 48%, depending on the patient’s heart rate.^23^

Myocardial viability can be assessed with different MSCT techniques. Most of them are based on techniques that were developed for MR imaging of myocardial infarction, including the assessment of the left ventricular wall thickness, evaluation of myocardial perfusion and delayed enhancement imaging. Most commonly, an original CT technique, the detection of hypo-enhancing myocardium during arterial phase, is used to detect myocardial infarction.

## Arterial-phase imaging

Contrast enhancement of the myocardium during arterial-phase CT is directly related to myocardial perfusion. As myocardial infarction is associated with a lack of perfusion, it can be assumed that myocardium with reduced contrast enhancement during arterial-phase CT is related to coronary artery stenosis, occlusion of an epicardial coronary artery, obstruction of intramyocardial arterioles or chronic myocardial scar. This technique was developed in the 1970s^24^ and the early 1980s. Promising results from the application of single-slice CT^25^,^26^ or EBCT for the detection of myocardial infarction have been reported.^27^ At that time, this technique was hampered by the inability to acquire short-axis images of the left ventricle, limiting the detection of inferior wall changes. This was the key limitation obviating the routine use of arterial-phase CT for diagnosing myocardial infarction.

**Fig. 1.**
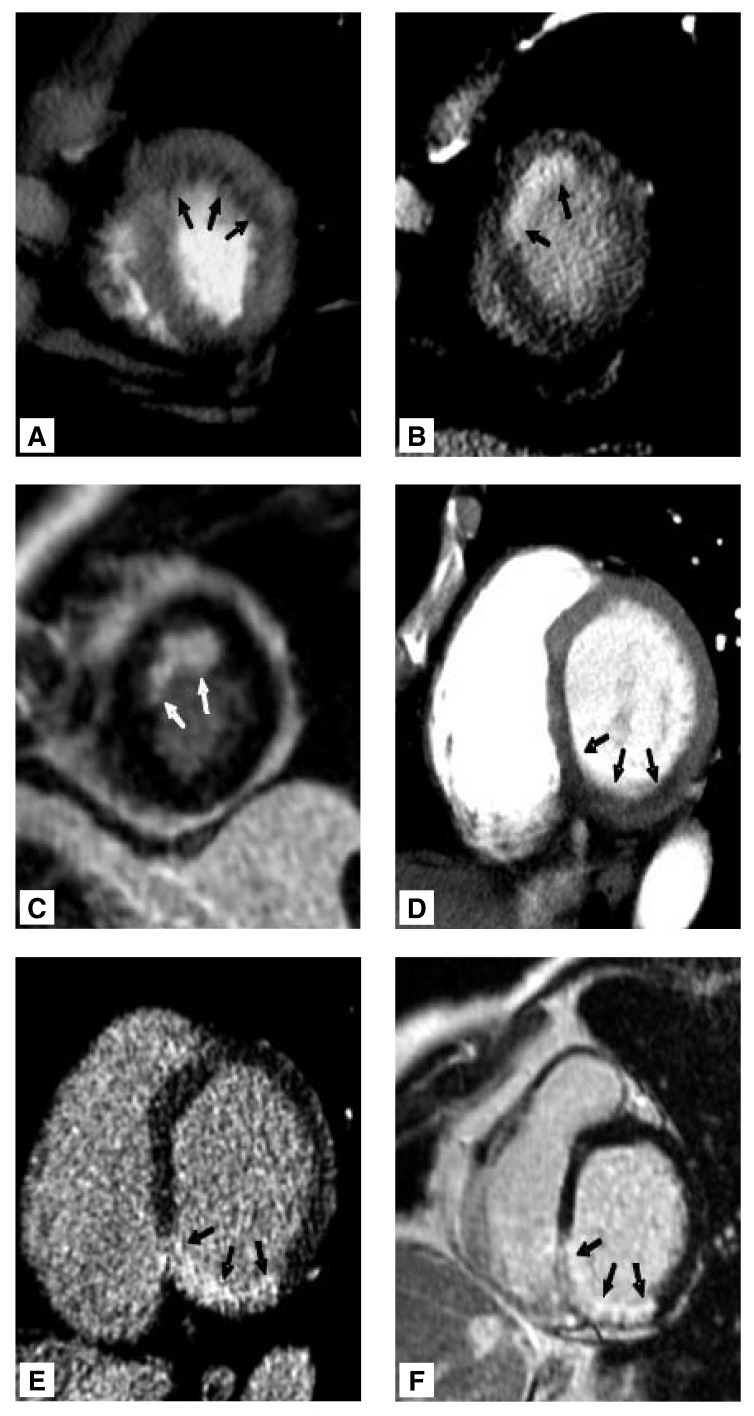
Arterial-phase CT depicts myocardial infarction as area of reduced contrast enhancement (A, D), while late-phase CT shows a delayed enhancement in the area of infraction (B, E) (arrows). CT can differentiate subendocardial (A−C) from transmural infarction (D−E). Extent and location of actual infarction on delayed enhanced CT (B, E) correlate better with MR imaging (C, F) than arterial-phase CT (A, D).

The introduction of MSCT dramatically changed the role of cardiac CT. With gantry rotation times down to 0.33 s, submillimetre spatial resolution and simultaneous development of advanced post-processing technologies, cardiac CT became popular again. As a consequence, acquisition of high-resolution MSCT angiography data allowed calculation of short-axis images and consequently, sufficient assessment of the inferior wall of the left ventricle. Recently, sensitivities and specificities ranging from 79 to 91% were reported for the detection of myocardial infarction by coronary MSCT when compared with ventriculography and MR imaging (Fig. 1; Table 2).^28^,^29^ Even non-gated chest CT led to similar results.^30^ Detection of wall motion abnormalities in the same MSCT data set supports the presence of myocardial infarction.^31^,^32^

**Table 2:** Several Studies Proved The Detection Of Myocardial Infarction Feasible From ECG-Gated Arterial Phase CT

*Author and reference*	*Patients with/without MI*	*Reference*	*Sensitivity (%)*	*Specificity (%)*
Nikolaou 2004^28^	27/106	Clinical	85	91
Nikolaou 2005^29^	11/30	MR imaging	91	79
Mahnken 200^58^	110/448*	MR imaging	83	91
Francone 2006^33^	29/187	Clinical	83	91
Sanz 2006^56^	21/42	MR imaging	91	81

* Segment-based analysis; MI = myocardial infraction; HU = Hounsfield units.

One main limitation is that reduced contrast enhancement during arterial phase not only represents myocardial infarction, but also reduced perfusion. As a consequence, reduced contrast enhancement can be found in viable as well as non-viable myocardium. Moreover, the size of infarction is significantly underestimated in arterial-phase CT.^8^ Although acute and chronic myocardial infarction both present with decreased myocardial attenuation during arterial phase, they may be distinguished by the wall thickness, as chronic myocardial infarction typically presents with myocardial wall thinning due to scar formation.^28^,^33^ The presence of decreased attenuation values in combination with reduced wall thickness is therefore likely to represent chronic myocardial infarction. The inability to differentiate non-viable acute myocardial infarction from viable hypoperfused myocardium limits the use of this technique in clinical routine.

The use of arterial-phase stress adenosine MSCT was demonstrated by a small single-centre study.^34^ With an agreement of 83%, the results correlated well with stress thallium-201 myocardial perfusion scintigraphy. This technique may help to differentiate acute myocardial infarction from reversible ischaemic damage of the myocardium. However, this approach is limited due to the repeated exposure to radiation and contrast material and has to be considered experimental.

## Perfusion imaging

To assess the significance of a coronary artery lesion, techniques to quantify the physiological relevance are needed. First-pass myocardial perfusion imaging is suited to address this problem as decreased myocardial perfusion represents the first consequence of obstructive coronary artery disease.^35^ This technique allows the detection of impaired microvascular function. Several studies have demonstrated electron beam computed tomography (EBCT), a non-invasive technique to evaluate intramyocardial microcirculatory function and to assess microvascular blood volume distribution.^36^ Recently, contrast-enhanced MSCT has also been shown to provide information about myocardial perfusion.^37^

Animal studies have shown the MSCT assessment of differences in myocardial perfusion to be feasible. For this purpose, rest and stress perfusion scanning were combined.^38^ Theoretically, coronary perfusion reserve can also be derived from MSCT data and blood-flow quantification becomes accessible.^39^ However, there is little data on this issue and MSCT perfusion techniques have not yet been implemented in clinical settings.

When compared with MR imaging, MSCT perfusion imaging requires radiation exposure, but also offers the advantage of the linear relationship between contrast enhancement and iodine concentration.^40^ In theory, this relationship permits the direct quantification of myocardial blood flow, omitting the need for potentially error-bearing correction methods.^41^ A key restriction is the limited examination range that is determined by the detector width.

## Delayed enhancement

To overcome the limitations of arterial-phase CT, other CT techniques were developed to selectively depict infarcted myocardium. For CT, it was shown as early as 1978 that extracellular contrast material accumulates in areas of acutely infarcted myocardium.^42^ With late-phase CT imaging, five to 20 minutes after injection of iodinated contrast agents, this can be visualised. During the early 1980s, several studies investigated the use of delayed enhancement for assessment of myocardial infarction with EBCT.^43^ This technique has so far not been valued as a clinical tool.^44^

This assessment changed with the introduction of MSCT. Recently, several studies in animals (Table 3) as well as in patients (Table 4) proved the reliability of delayed enhanced MSCT in comparison with MR imaging, SPECT and pathological assessment.^8^,^45^-^47^ As the molecular weight and volume of distribution of extracellular MR and CT contrast agents are almost identical, very similar contrast dynamics and enhancement patterns were observed.^39^ Due to the high spatial resolution of MSCT, even subendocardial infarctions can be detected (Fig. 1). Detection of microvascular obstruction with its prognostic implications is also feasible.

**Table 3:** Animal Studies Proving Late-Phase MSCT Feasible For Assessing Myocardial Viability

*Author and reference*	*Animal*	*MI age*	*MI size MSCT (%)*	*MI size MR imaging (%)*	*MI size TTC (%)*
Buecker 2005^49^	14 pigs	Acute	22.8 ± 9.2	20.8 ± 11	20.6 ± 12
Baks 2006^57^	10 pigs	Acute	21 ± 15	22 ± 16	20 ± 15
Lardo 2006^58^	10 dogs	Acute	21.4	–	20.8
Lardo 2006^58^	7 pigs	Chronic	4.2 ± 1.9	–	4.9 ± 2.1

MI = myocardial infarction.

**Table 4:** Patient Studies Comparing MSCT With Spect Or MR Imaging In Acute Myocardial Infarction

*Author*	*Patient/segment*	*Reference*	*Sensitivity (%)*	*Specificity (%)*
Paul 2005^46^	34/578	SPECT	78	91
Mahnken 200^58^	28/448	MR imaging	97	98
Gerber 2006^47^	16/256	MR imaging	85	90

On delayed enhanced MSCT, myocardial infarction shows increased attenuation values when compared with healthy myocardium. No-reflow areas present as hypodense regions surrounded by hyperenhanced myocardium.^48^ Moreover, the ability to differentiate occlusive from reperfused myocardial infarction has been shown.^49^ While occlusive myocardial infarction presents up to one hour later as areas of diminished attenuation after injection of contrast material, reperfused myocardial infarction presents with the typical delayed enhancement. The non-enhancement in non-reperfused infarcted myocardium is attributed to a lack of collaterals and consequently lack of contrast material inflow to the area of infarction.

In general, delayed enhanced MSCT slightly overestimates the size of infarction. This might be due to an increase of the distribution volume in the peri-infarction zone.^50^ In the assessment of chronic myocardial infarction with a follow-up of up to three months, a decrease in infarct size suggests shrinkage of the damaged area, with subsequent fibrosis.^51^ Myocardial late enhancement, however, is not specific for myocardial infarction. As is well known from MR imaging, it can be found in several other cardiac pathologies, eg, sarcoidosis.^52^

So far, there is no unequivocal agreement on the most suitable protocol for delayed enhanced CT imaging. One of the major issues is the mode of contrast application and the optimal delay to image acquisition. Some studies showed best contrast between infarcted and normal myocardium at five minutes after injection.^47^ These findings are also supported by studies using MR imaging.^53^ As contrast delivery to non-viable tissue is a time-dependent process that will take several minutes to be completed, this was to be expected.

**Fig. 2.**
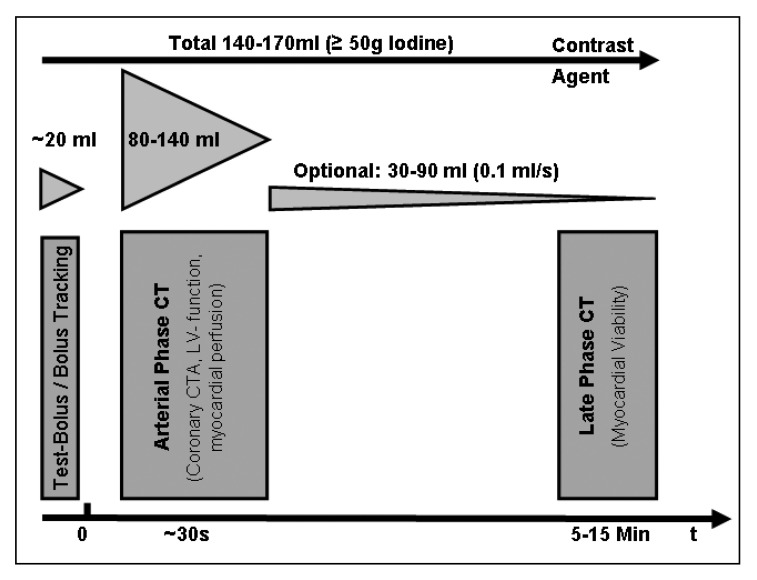
For a comprehensive work-up in patients with suspected or known myocardial infarction a dual-phase examination is recommended. There are no recommendations with respect to the mode of contrast injection and delay.

When compared to MR imaging, delayed enhanced MSCT imaging of myocardial infarction presents low contrast between the area of infarction and the blood pool. While a delay of several minutes is needed to allow contrast material to accumulate in infarcted myocardium, rapid fading of contrast is a major problem for accurate delineation of myocardial infarction with MSCT. Consequently, selection of an optimal delay is crucial for late enhanced CT images, with suggested intervals ranging from five to 15 minutes. Moreover, dedicated contrast injection protocols have to be developed to optimise contrast. The total amount of contrast material should exceed 50 g of iodine to ensure a sufficient contrast on late enhanced MSCT images. So far, there is no consensus whether a continuous injection of contrast material is superior to a single bolus injection for improving the quality of late-phase MSCT images (Fig. 2).

A different approach to improve contrast in delayed enhanced CT is the application of 80 kVp protocols that will result in better iodine contrast at the cost of greater image noise.^54^ When applying this technique, more subtle differences in attenuation can be detected.

When combining arterial- and late-phase MSCT, different contrast enhancement patterns can be observed. In some patients with delayed enhancing myocardium, an area of reduced attenuation might be present during arterial phase. According to Koyama *et al.* the lack of a hypo-enhanced area during arterial phase may indicate successful reperfusion at both the epicardial and the microvascular level.^48^

The difference in size between arterial and late enhanced CT in a rabbit animal model was considered to reflect coronary reperfusion.^55^ Consequently, a dual-phase scan protocol is needed for a comprehensive assessment of patients with suspected or known history of myocardial infarction. Ventricular volumes and regional wall motion are assessed from arterial-phase images, while myocardial viability is assessed from contrast-enhanced late-phase MSCT images. For predicting the individual patient’s outcome, arterial- and late-phase MSCT images are needed.^48^

## Conclusion

MSCT allows for the reliable assessment of myocardial viability. In patients with contra-indication to MR imaging, or if MR imaging is not available, MSCT appears to be the alternative of choice for the diagnostic work-up in patients with myocardial infarction. In combination with non-invasive coronary MSCT angiography and analysis of left ventricular function, delayed enhanced MSCT offers a comprehensive examination strategy for evaluation of the heart.
